# Supplementary material for: Lactobacilli Infection Case Reports in the Last Three Years and Safety Implications
Source: Nutrients. 2022 Mar 11;14(6):1178. doi: 10.3390/nu14061178 (PMC8954171; doi:10.3390/nu14061178)
Supplement: Supplementary file 1 [file nutrients-14-01178-s001.zip › nutrients-1624733-supplementary.pdf]

Table S1. Published case reports of infections caused by lactobacilli since 2019 with indication of age of the patient, underlying conditions, species identity of the etiological agent and identification method.

Type of infection	Age, sex	Risk factors	Underlying conditions	Aethiological agent	Reference
			**2019**		
Bacteremia	Neonate	preterm birth<br>treatment with *L. reuteri* probiotic		*Limosilactobacillus reuteri*	[20]
	46 male	none	diabetes mellitus	unidentified lactobacilli	[21]
	62 male	treatment with Nivolumab	lung cancer	*L. acidophilus*	[22]
	46 female	urinary tract infection (UTI), polysubstance abuse	diabetes mellitus	*L. acidophilus**	[23]
	Neonate female	preterm birth<br>peripherally inserted central catheter (PICC) and administration of Dicoflor®		*L. rhamnosus**	[24]
	2 neonates, males	PICC, use of Dicoflor® in the same room		*L. rhamnosus**	[24]
Endocarditis	39 male	bioprostetic aortic valve; intravenous drug abuse		unidentified lactobacilli	[14]
	65 male	bioprostetic aortic valve; dental extraction		*L. paracasei**	[15]
	75 female	bioprostetic aortic valve	Birt-Hogg-Dube syndrome	*L. paracasei*	[16]
	75 female	septic shock from acute cholecistitis; prostetic aortic valve	Birt-Hogg-Dube Syndrome	*L. paracasei*	[17]
	74 male	bioprostetic aortic valve; probiotic consumption		unidentified lactobacilli	[18]
	72 male	prosthetic valve	diabetes mellitus; stage II chronic kidney disease; coronary artery disease	unidentified lactobacilli	[19]
Meningo-encephalitis	63 male			*L. plantarum*	[46]
Septic shock	54 male	consumption of probiotic yogurt	promyelocytic leukemia in second complete remission	*L. rhamnosus* GG†	[47]
UTI	49 male		none	*L. delbrueckii* subsp. *delbruekii*	[48]

Perinephric abscess	52 male		diabetes mellitus, obesity, mild hydronephrosis	unidentified lactobacilli	[49]
			**2020**		
Bacteremia	75 male	dental scaling; immunosuppression for renal transplantation	diabetes mellitus	*L. rhamnosus*‡	[35]
Endocarditis	42 male	bioprosthetic aortic valve; history of IV drug use	diabetes mellitus	*L. acidophilus*	[25]
	48 male	bioprosthetic valve		*L. plantarum*	[26]
	57 male	teeth scaling one year earlier, heart surgery in 2003		*L. rhamnosus**	[27]
	50 male	gingival laceration, probiotic use	possible undiagnosed structural heart disease	*L. rhamnosus**	[28]
	60 male	dental caries		*L. acidophilus*	[29]
	45 male	consumption of probiotic yogurt	gastroesophageal reflux	*L. paracasei*†	[30]
	57 female	asymptomatic ureteric obstruction by calculi	diabetes mellitus, hypertension	*L. jensenii*	[31]
	60 male	none	diabetes mellitus, Erdheim-Chester disease on chemotherapy	*L. rhamnosus*	[32]
	40 male	past use of illicit drugs; smoking; poor dentation and caries	diabetes mellitus	*L. rhamnosus*	[33]
	83 male	prosthetic valve; recent upper endoscopy	pancytopenia; cirrhosis; Crohn's disease	Unidentified lactobacilli	[34]
Interstitial pneumonia	68 female	*L. paraplantarum* probiotic supplementation	pancreatic cancer; diabetes mellitus	No cultures were carried out	[50]

Lung abscess	14 male	possible aspiration of lactobacilli from yogurt	cerebral palsy, epilepsy and asthma treated with corticosteroids	*L. rhamnosus*	[51]
Renal and perinephric abscesses	26 female	interventions to treat nephrolithiasis		*L. jensenii** (association with *Prevotella bivia*)	[52]
Prosthetic joint infection	82 female	hip arthroplasy	nephrectomy 16 years earlier, asthma, hypertension, dyslipidemia and hypothyroidism	*L. paracasei*	[53]
			**2021**		
Bacteremia	35 male	consumption of self made yogurt	Crohn's disease, HIV-infection	unidentified lactobacilli	[42]
	72 female	consumption of Nukazuke, a type of fermented vegetable preparation	mild hypertension, colon adenocarcinoma	*L. plantarum**‡	[43]
	Neonate male	treatment with a probiotic	aortic coarctation	*L. rhamnosus* GG (ATCC 53103)	[44]
	Neonate female	pre-term birth, CVC, treatment with a probiotic none		*L. rhamnosus* GG (ATCC 53103)+ 45. Chiang et al. (2021)	[45]
Endocarditis	47 male		cardiac disease	*L. paracasei* LP10266+	[36]
	81 male	transcatether aortic valve implantation		*L. rhamnosus**‡	[37]
	83 female			*L. rhamnosus**‡	[37]
	55 female		bicuspid aortic valve	*L. jensenii*	[38]
	47 male			unidentified lactobacilli	[39]
	40 male	mitral valve repair	none	*L. jensenii**+	[40]
	71 male	recent coronary artery stent placement	hypertension; obstructive sleep apnoea	*L. rhamnosus*	[41]
Masticator abscess	23 female	wisdom tooth extraction,	diabetes mellitus	unidentified lactobacilli	[54]
Prostatic abscess	57 male		diabetes mellitus hypertension acute renal disfunction	*L. jensenii*‡	[55]
Liver abscesses	59 male	multiple abdominal surgeries with modified biodigestive anatomy	diabetes mellitus	*L. gasseri**	[56]

Pancreatic necrosis and retroperitoneal abscess	88 female	diabetes mellitus, hypertension	*L. paracasei*	[57]
Cavernosal abscess	63 male	diabetes mellitus	*L. gasseri** *L. paragasseri*‡	[58]

*identified by MALDI TOF mass spectrometry and/or standarzized colorimetric methods;
†identified by PCR;
‡identified by 16S rRNA gene sequencing;
+identified by genome sequencing.
